# Systemic Vascular Function Is Associated with Muscular Power in Older Adults

**DOI:** 10.1155/2012/386387

**Published:** 2012-08-26

**Authors:** Kevin S. Heffernan, Angela Chalé, Cynthia Hau, Gregory J. Cloutier, Edward M. Phillips, Patrick Warner, Heather Nickerson, Kieran F. Reid, Jeffrey T. Kuvin, Roger A. Fielding

**Affiliations:** ^1^Human Performance Laboratory, Department of Exercise Science, Syracuse University, Syracuse, NY 13244, USA; ^2^Nutrition, Exercise Physiology and Sarcopenia Laboratory, Jean Mayer USDA Human Nutrition Research Center on Aging at Tufts University, Boston, MA 02111, USA; ^3^The Vascular Function Study Group, Division of Cardiology and the Molecular Cardiology Research Institute, Tufts Medical Center, Boston, MA 02111, USA

## Abstract

Age-associated loss of muscular strength and muscular power is a critical determinant of loss of physical function and progression to disability in older adults. In this study, we examined the association of systemic vascular function and measures of muscle strength and power in older adults. Measures of vascular endothelial function included brachial artery flow-mediated dilation (FMD) and the pulse wave amplitude reactive hyperemia index (PWA-RHI). Augmentation index (AIx) was taken as a measure of systemic vascular function related to arterial stiffness and wave reflection. Measures of muscular strength included one repetition maximum (1RM) for a bilateral leg press. Peak muscular power was measured during 5 repetitions performed as fast as possible for bilateral leg press at 40% 1RM. Muscular power was associated with brachial FMD (*r* = 0.43, *P* < 0.05), PWA-RHI (*r* = 0.42, *P* < 0.05), and AIx (*r* = −0.54, *P* < 0.05). Muscular strength was not associated with any measure of vascular function. In conclusion, systemic vascular function is associated with lower-limb muscular power but not muscular strength in older adults. Whether loss of muscular power with aging contributes to systemic vascular deconditioning or vascular dysfunction contributes to decrements in muscular power remains to be determined.

## 1. Introduction

 As life expectancy in the United States continues to rise, the maintenance of physical independence of older adults has also emerged as a major clinical and public health priority. A critical factor in an older person's ability to function independently is the ability to move without assistance. Older adults who lose mobility are less likely to remain in the community, have higher rates of mortality, and experience a poorer quality of life [1, 2]. Age-associated loss of muscular strength (the ability to generate maximal muscle force) and muscular power (the product of the force and velocity of muscle contraction) is an important determinant of this loss of physical function and progression to disability [3]. Interestingly, although muscular strength and power are associated, muscular power has been shown to be a stronger predictor of physical function than muscular strength in older adults [4, 5]. Poor muscular power is associated with a 3-fold greater risk for mobility impairment than poor muscle strength [6] and improving muscular power leads to improvements in physical function independent of changes in muscular strength [7]. 

Although numerous potential mechanisms have been put forth, no single common etiology is believed to be responsible for age-associated loss of muscular strength/power and progression to disability. In many cases, physical disability is directly caused or aggravated by cardiovascular disease (CVD) or chronic CVD-related conditions (heart failure, coronary heart disease, and diabetes) [8–10]. Vascular senescence is a phenotypic expression of human aging that contributes to increased CVD risk [11, 12]. With advancing age, there is pervasive macrovascular and microvascular dysfunction that manifests as stiffening of central elastic arteries, elevation of pressure from wave reflections, and peripheral endothelial dysfunction [11, 12]. An ever-growing literature now supports the notion that vascular function may affect physical function.

Several cross-sectional associations between metrics of vascular function and metrics of functional reserve have been noted [13–16]. Nitric oxide (NO) is a potent vasoactive metabolite released by endothelial cells that helps regulate vascular tonus. Age-associated reduction in NO contributes importantly to the development of endothelial dysfunction and vasomotor dysfunction [17]. This may affect physical function by reducing blood flow into the active capillary bed and curtailing efficient nutrient transport (e.g., oxygen, lactate, pyruvate) [18]. To date, most studies that have explored associations between vascular function and physical function have examined activities that are more aerobic in nature (long distance gait performance, exercise capacity/endurance, etc.). Few studies have explored the association between vascular function and measures of physical function that are of anaerobic origin. Separate from its effects on blood flow and tissue perfusion, NO also plays a crucial role in maintaining optimal skeletal muscle contractile function in vitro. NO enhances velocity of muscle fiber shortening, reduces twitch time-to-peak contraction, and increases rate of force development via modulation of mechanical and metabolic muscle fiber machinery [19]. Conversely, inhibition of NO significantly reduces skeletal muscle maximal force generating capacity and increases muscular fatigue [20]. Thus loss of NO bioavailability may impact skeletal muscle function (force and velocity), manifesting as reductions in muscular power and strength in vivo although this has yet to be explored.

The purpose of this study was to test the hypothesis that NO-mediated vascular reactivity (brachial artery flow-mediated dilation and digital pulse wave amplitude reactive hyperemia index) and systemic vascular function related to arterial stiffness/wave reflections (appraised via the augmentation index) is associated with muscular strength and muscular power in older adults with mobility limitations.

## 2. Methods

Twenty-four men and women (*n* = 13) between the ages of 70 and 85 years with mobility limitations were recruited from the greater Boston community to participate in this study. Individuals “at risk for mobility disability” were defined as scoring ≤10 on the Short Physical Performance Battery as previously described [21] and discussed below. Exclusion criteria were as follows: acute or terminal illness, myocardial infarction in the previous 6 months or other symptomatic coronary artery disease, upper or lower extremity fracture in the previous 6 months, uncontrolled hypertension (>150/90 mmHg), neuromuscular diseases and drugs which affect neuromuscular function, hormone replacement therapy, type 2 diabetes mellitus, renal disease (eGFR <60 mL/min/1.73 m^2^), and body mass index (kg/m^2^) <21 or >32.5. All participants gave written informed consent and this study was approved by the Tufts University Health Sciences Campus Institutional Review Board. 

## 3. Study Design 

All hemodynamic and vascular measures were carried out in the early morning with the participant in a fasted state (>12 postprandial). Participants on medication with known vasoactive properties were instructed to refrain from taking these medication(s) for 5 times the half-life of the agent (calculated individually per medication). Participants were required to rest quietly in a supine position for a minimum of 15 minutes in a dimly lit, climate control room prior to all measures. Blood pressure was measured in triplicate. This was followed by assessment of vascular function with ultrasound and concurrent measures of PAT. On a separate day prior to vascular assessment (within 3 days) physical mobility was assessed using the SPPB. Muscular strength and muscular power were determined using a pneumatic leg press machine. Finally, physical activity was assessed from 7-day accelerometry recordings. 

### 3.1. Blood Pressure

 Blood pressure was measured using a validated automated device (Dynamap, GE Healthcare, USA). Pulse pressure was calculated as systolic blood pressure (SBP)-diastolic blood pressure (DBP). Mean arterial pressure (MAP) was calculated as 1/3 SBP × 2/3 DBP. Following measurement of blood pressure, measures of vascular endothelial function were carried out. 

### 3.2. Flow-Mediated Dilation

 The brachial artery was longitudinally imaged 2 cm above the antecubital fossa using a 10 mHz linear array vascular ultrasound transducer and portable ultrasound unit (SonoSite Titan, Bothell, WA,USA) as previously validated by our group [22]. Diameters were measured during end diastole (gated with ECG R-waves) using ultrasonic calipers (the average of 5 evenly spaced measures along the lumen border) at baseline and 60 seconds after release of a blood pressure cuff around the upper arm (inflated to 200 mmHg for 5 minutes). Responses were calculated as percentage change in brachial artery diameter from baseline (flow mediated dilation, FMD). Mean intraobserver and interobserver variability of brachial reactivity measurements in our laboratory on normal volunteers were previously shown to be 1.9% and 2.8%, respectively [23]. Heart rate was computed from the aforementioned ECG recordings.

### 3.3. Reactive Hyperemia Index and Augmentation Index

Specially designed finger pneumatic sensors (EndoPAT, Itamar Medical Ltd, Israel) were used to continuously record pulsatile volume changes (measured as pulse wave amplitude) in the finger as previously validated by our group [24]. This was done before and during reactive hyperemia induced by brachial artery circulatory occlusion for 5 minutes as described previously. The pulse wave amplitude (PWA) reactive hyperemia index (RHI) is calculated as the ratio of the average PWA over a 1-minute epoch starting 60 seconds after 5 min of ischemia induced by brachial cuff inflation to a suprasystolic BP, divided by the average PWA of a 3.5-minute baseline epoch. A finger probe is also placed on the contralateral hand in order to capture any systemic changes that might occur during local reactive hyperemia. The signal is amplified, filtered, and stored in a computer for analysis. Diminution of PWA-RHI is suggestive of resistance vessel endothelial dysfunction. 

 From the digital pulse volume waveforms, peak volume and inflection points were identified using a 4th-order derivative computerized algorithm as previously described by Kelly et al. [25] and Takazawa et al. [26]. AIx was calculated as the ratio of the difference between the early and late systolic peaks of the waveform relative to the early peak expressed as a percentage. Previous studies have noted that the association of AIx derived from PAT and AIx derived from synthesized aortic pressure waves is comparable [27, 28]. Reproducibility of these techniques has previously been shown to be moderately high (ICC 0.5–0.8 with between day variability of approximately 11%) [29–31]. 

### 3.4. Physical Function

Participants completed the short physical performance battery (SPPB) which consists of the following tasks: timed short distance walk, repeated chair stands, and a balance test. Walking speed was assessed by asking the participants to walk at their usual pace over a 4 m course. Two trials were conducted and the faster of 2 used for subsequent analyses. The repeated chair stands test was performed by asking participants to first stand from a sitting position without using their arms. If they were able to perform the task, they were then asked to stand up and sit down 5 times, as quickly as possible. The time to complete the task was recorded. For the test of standing balance, participants were asked to maintain balance in 3 positions, characterized by a progressive narrowing of the base support: feet together (side by side position), the heel of one foot beside the big toe of the other foot (semi tandem position), and the heel of one foot in front of and touching the toes of the other foot (tandem position). For each of the 3 positions, participants were timed to a maximum of 10 sec. Each of the 3 performance measures was assigned a score ranging from 0 to 4, with 4 indicating the highest level of performance and 0 the inability to complete the test. A summary score ranging from 0 (worst performers) to 12 (best performers) was calculated by adding walking speed, chair stands, and balance scores. This scale has proven reliable [32] and valid for predicting institutionalization, hospital admission, mortality, and disability [33]. 

### 3.5. Muscular Strength and Power

One repetition maximum (1RM, greatest load that could be moved one time through the full range of motion using proper technique) was determined for a bilateral leg press using a pneumatic machine (K400, Keiser Sports Health Equipment Inc, Fresno, CA), designed to provide variable resistance via utilization of attached cylinders pressurized with air (i.e., compression of gas within affixed cylinder produces resistance). Range of motion was established by having the participant fully extend the dominant limb against minimal resistance. This position was recorded in real time. 1RM attempts were considered successful if the participant reached 90% of the established criterion limb position. Resistance was increased after each trial unless the participant failed to achieve 90% of maximal limb position, in which case the resistance was reduced by half of the previous increment. Testing continued until the participant failed two consecutive trials at a given level of resistance. Each trial was followed by 1 min of rest. Testing was carried out twice, separated by one week. The 1 RM was established as the best value from the two trials due to the well-established learning effect of this measure and this was typically attained within 6–8 trials. For the assessment of muscular power, all participants performed 5 repetitions as fast as possible for bilateral leg press at 40% 1RM. Five maximal effort trials were performed, with at least 30 s of rest between each trial. Software developed by the manufacturer calculates power during the concentric phase of each repetition by analyzing system pressure (force) and leg press platform displacement at a sampling frequency of 400 samples/second. The highest power output achieved during each of these 5 repetitions (data collected between 5% and 95% of the concentric phase in order to minimize signal noise) was taken as peak power/velocity. The reliability of these measurements in older subjects in our group is high (leg press ICC = 0.95) [34].

### 3.6. Physical Activity

Accelerometry allows objective measurement of physical activity by the use of a motion sensor which records both the number and magnitude of vertical accelerations generated by human movement and was performed using the Actigraph Model 7164 (Manufacturing Technology Inc., FL, USA). The Actigraph was worn superior to the iliac crest in a custom pouch, secured to the participant's belt by a Velcro fastening. Participants were instructed to wear the Actigraph for a 7-day period during waking hours and remove it for sleep and bathing only (confirmed with activity logs). Activity was recorded using 1 min epochs and a valid day of measurement was defined as >10 h of wear with <1 h of no accelerometer data (i.e., continuous zeros). Total daily movement counts were calculated by summing the minute-by-minute counts across each 24 h epoch and then averaging across valid days of measurement. Using this criterion, 4 participants did not have complete 7-day physical activity measures. All participants had 6 valid days of activity data; therefore daily activity was derived from 6 days. 

### 3.7. Statistical Analysis

 Normality of distribution was verified using Kolmogorov-Smirnov and Shapiro-Wilk tests. All data are reported as means ± standard error of the mean. A priori significance was set at *P* < 0.05. Pearson correlation coefficients were used to assess relationships between measures of physical function (1RM, peak power at 40% 1RM, physical activity counts, and SPPB score) and measures of vascular function (brachial FMD, PWA-RHI, and AIx). Given the potential confounding influence of sex, BMI, mean arterial pressure, and heart rate on measures of muscular strength/power and vascular function, partial correlations were used to examine associations between measures of physical function and vascular function after adjusting for these potential confounders. 

## 4. Results

**Figure 1:**
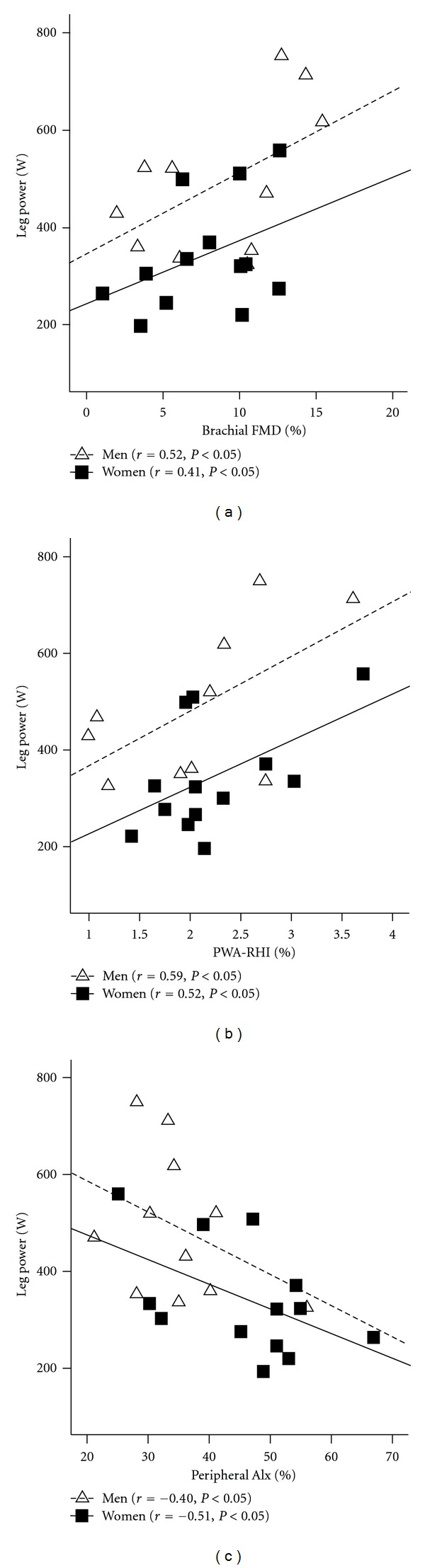
Association between leg power (assessed as 5 repetitions performed as fast as possible at 40% 1RM) and (a) brachial artery flow-mediated dilation (FMD); (b) pulse wave amplitude-reactive hyperemia index (PWA-RHI); (c) peripheral augmentation index (AIx). Open triangles with dashed line (men); closed squares with solid line (women).

 Participant descriptive characteristics are presented in Table 1. Muscular strength and power were significantly higher in men versus women (*P* < 0.05). Groups did not differ in age, CVD risk factors or medication use. As seen in Table 2 and displayed in Figure 1, muscular power was associated with brachial FMD (*P* < 0.05), PWA-RHI (*P* < 0.05), and AIx (*P* < 0.05). Muscular strength was not associated with these parameters. After adjusting for sex, BMI, MAP, and HR, associations between measures of muscular power and vascular function remained (Table 3, *P* < 0.05). Associations between muscular power and FMD (*r* = 0.59, *P* < 0.05), PWA-RHI (0.58, *P* < 0.05), and AIx (−0.42, *P* < 0.05) remained after adjusting separately for strength. 

## 5. Discussion

Muscle power has emerged as a critical determinant of impairment in older adults, independent of muscular strength. As such understanding factors that potentially limit muscle power and subsequently functional reserve in older adults have received considerable attention [35]. Vascular dysfunction with aging is associated with decreases in physical function and performance of activities of daily living [13, 36] alluding to the notion that the genesis and demise of muscular power may in part have a vascular basis. The novel findings from the present investigation were as follows. Our hypothesis was partially supported in that vascular endothelial function (appraised using two distinct techniques) and AIx were each associated with lower-limb muscular power in older adults. Contrary to our hypothesis, vascular function was not associated with lower-limb muscle strength. 

To date, most studies that have explored associations between vascular function and physical function have examined activities that are more aerobic in nature (long distance gait performance, exercise capacity/endurance, etc.). With aging and disease, limitations in oxygen delivery may limit contractile work and age-associated impairment in endothelial-dependent vasodilation contributes to lower exercise hyperemia [37, 38]. During exercise, vasodilation occurs via an increase in several vasoactive agents including but not limited to nitric oxide (NO) [39, 40]. Resultant increases in blood flow ensure that oxygen delivery is closely matched to metabolic demand. Endothelium-dependent vasodilation increases circulatory time in the capillaries and allows for more effective oxygen and nutrient extraction in the tissues [41]. NO synthase inhibition reduces exercise blood flow to active skeletal muscle [40] and with aging the relative contribution of NO to exercise hyperemia is reduced by approximately 45% [37]. A reduction in flow to working skeletal muscle (i.e., reduced perfusion) may ultimately limit the ability to perform functional tasks. Indeed with aging there is a generalized reduction in limb blood flow and vascular conductance both at rest [42] and during exercise contributing to reduced exercise capacity [43, 44]. 

Recently, we and others have reported associations between vascular function and more anaerobic processes (i.e., muscular strength) [45, 46] and studies are emerging noting associations between measures of vascular function and skeletal muscle morphology and muscle fiber type in middle-aged [47] older adults [48, 49]. Endothelial-dependent (predominantly nitric oxide mediated) vasodilation of peripheral conduit vessels can be assessed in vivo by examining the change in brachial artery diameter in response to an increase in shear stress induced by regional occlusion (flow mediated dilation, FMD)[50, 51] whilst endothelial-dependent (predominantly nitric oxide mediated) vasodilation of peripheral resistance vessels can be quantified similarly by examining the change in digital pulse wave amplitude during reactive hyperemia (peripheral arterial tonometry reactive hyperemia index, PAT-RHI) [52]. Moreover, augmentation index (a measure of systemic vascular function related to arterial stiffness and pressure from wave reflections) can also be influenced substantially by NO [53]. Using these techniques, our findings extend previous observations and note that vascular function is also a correlate of muscle power in older adults. 

Although the exact mechanism explaining preferential associations between vascular function and muscular power versus strength is not completely understood, we suggest that it may be related to loss of NO bioavailability. Age-associated loss of muscular power progresses at a faster rate then declines in muscular strength [54]. Selective loss of fast-twitch/type II fibers with aging may partially explain this observation as type II fibers have a peak power output 4 times that of type I fibers [55]. Of interest, NO has been shown to play a crucial role in satellite cell activation and subsequent hypertrophy of fast twitch fibers [56] and prevents muscle wasting in response to cachectic stimuli [57]. With aging, there appears to be redistribution of muscle blood flow during exercise from highly oxidative to highly glycolytic muscles and this is a nitric-oxide-mediated/endothelium-dependent process [58–60]. NO has been shown to play a pivotal role this phenotypic “slow-to-fast shift” [19]. Greater flow to fast-twitch glycolytic fibers may be a compensatory adaptation aimed at preserving contractile function concomitant with reduced fiber volume. Indeed recent studies note that, despite age-associated changes in muscle mass, single-fiber contractile properties of type II fibers are preserved [61, 62]. 

Type I oxidative fibers have severalfold greater blood flow than type II glycolytic fibers and NO inhibition drastically blunts blood flow to oxidative fibers [63, 64]. Thus flow to oxidative fibers also appears to be under endothelial control [60]. Although peak power development of type II fibers may not change with aging, peak power of type I fibers decreases [61]. NO has been shown to optimize shortening velocity of slower fibers without influencing faster fibers [65]. This is important as velocity (in addition to force) is a significant determinant of muscle power in older adults in vivo [35]. With aging, there is increased oxidative stress which reduces NO bioavailability resulting in formation of peroxynitrite in vascular and skeletal muscle [66, 67]. Peroxynitrite has been shown to decrease contractile force in slow-twitch fibers with little effect on fast-twitch muscle fibers [68]. It is important to note that the size principle still prevails during maximal contractions. Thus initial recruitment of and perfusion to these type I fibers is an important component of the overall genesis of muscular power. NO has also been implicated in numerous aspects of excitation contraction uncoupling with aging (reduced sarcoplasmic reticulum calcium-ATPase activity, reduced ryanodine receptor-mediated calcium release, reduced calcium sensitivity) [67, 69, 70]. Endothelial dysfunction and loss of NO bioavailability may drastically reduce perfusion to type I fibers and directly impact fiber contractile characteristics of type II fibers, reducing overall force generating capacity and peak power development [19]. Taken together these findings suggest that NO is needed to maintain optimal contractile function (notably velocity) of both slow-twitch and fast-twitch fibers and this may explain preferential associations between NO-mediated measures of vascular reactivity and muscular power in vivo. 

There is a bidirectional relationship between pressure from wave reflections (AIx) and NO bioavailability. Each cardiac contraction generates an outgoing pressure wave that traverses the systemic vasculature. Encountering areas of impedance both structural (i.e., bifurcations) and function (i.e., vasoconstricted vessel), these pressure waves are reflected back toward their origin. AIx assessed in the digital circulation is influenced by wave reflections originating in the hand microvasculature as well as the lower body [71]. NO blockade has been shown to increase AIx likely via altering vascular tone which may alter timing and magnitude of reflected pressure waves [53]. Pressure from peripheral wave reflections augment central pressure but subtract from flow. This reduces antegrade flow into the active vascular bed and increases retrograde flow in upstream conduit vessels which causes endothelial dysfunction and loss of NO bioavailability [72, 73]. Indeed retrograde flow increases with age and is associated with reduced physical function [36]. 

Given the cross-sectional nature of this study, direct causality cannot be determined, only inferred. Thus alternative interpretations should be considered. Mobility limitations perpetuate a sedentary lifestyle. This is important as physical inactivity is a CVD risk factor and a potent instigator of vascular maladaptation [74, 75]. Within 4-5 days of inactivity induced by bed rest there are reductions in limb blood flow [76] and signs of endothelial dysfunction [77]. As little as 2 weeks of limb immobilization has been shown to increase arterial stiffness [78]. Thus immobility in the elderly may perpetuate inactivity and reduce muscle power further promulgating a state of “extreme” vascular deconditioning. 

A limitation to this study is the relatively small sample (i.e., examination of several correlations may increase chances of a type II statistical error). However, the observation that three* independent* measures of vascular function were each associated with muscular power lends support to the objectivity of overall findings. Although vascular measures have overlapping physiologic underpinnings, the direct association between techniques is nonexistent to weak at best [71, 79, 80]. Indeed recent large-scale studies note that each measure is associated with different measures of vascular risk and endothelial biology [81–83] suggesting that each provides slightly different insight into vascular physiology in vivo.

In conclusion, vascular function is associated with muscular power in older adults. This association remains after adjusting for important confounders such as sex, BMI, and muscular strength. Future research is needed to examine if improving vascular function and/or preserving NO bioavailability leads to concomitant improvements in muscular power and prevention of physical impairment in older adults. 

## Figures and Tables

**Table 1:** Participant characteristics (*n* = 24).

Variable	All	Women (*n* = 13)	Men (*n* = 11)
Age, yrs	78 ± 1	77 ± 1	80 ± 1
Body mass index, kg/m^2^	26 ± 1	26 ± 1	26 ± 1
Systolic blood pressure, mmHg	130 ± 3	130 ± 4	130 ± 4
Diastolic blood pressure, mmHg	74 ± 1	74 ± 3	75 ± 3
Mean arterial pressure, mmHg	93 ± 2	94 ± 2	93 ± 2
Heart rate, bpm	62 ± 2	63 ± 3	61 ± 2
Total cholesterol, mg/dL	177 ± 6	184 ± 8	163 ± 10
LDL cholesterol, mg/dL	105 ± 4	109 ± 6	100 ± 8
HDL cholesterol, mg/dL	54 ± 3	58 ± 3	46 ± 4*
Triglycerides, mg/dL	96 ± 9	107 ± 16	84 ± 5
Glucose, mg/dL	93 ± 2	94 ± 3	92 ± 3
White blood cell count	5.0 ± 0.3	5.2 ± 0.4	4.9 ± 0.3
Neutrophil/lymphocyte ratio	1.9 ± 0.1	1.8 ± 0.2	2.0 ± 0.2
Hyperlipidemia, %	33	31	36
Hypertension, %	50	38	62
Medications, %			
Aspirin	33	31	36
ACE inhibitor	26	15	36
Beta-blocker	25	18	31
Calcium channel blocker	17	8	27
Diuretic	25	23	27
Statin	33	31	36
Leg press 1 RM	1237 ± 77	1040 ± 76	1470 ± 107*
Leg press peak power 40% 1 RM	417 ± 30	351 ± 33	491 ± 46*
SPPB, score	8.5 ± 0.5	8.3 ± 0.4	8.9 ± 0.3
Physical activity, counts	93224 ± 14465	103072 ± 22766	81586 ± 17023
Brachial FMD, %	7.5 ± 0.8	8.8 ± 1.0	7.1 ± 1.0
PWA-RHI, %	2.1 ± 0.2	2.3 ± 0.1	1.8 ± 0.3
AIx, %	40 ± 3	46 ± 3	35 ± 3*

^∗^Significant group difference, *P* < 0.05.

**Table 2:** Correlation matrix for measures of vascular function and muscle function.

	40% 1 RM	1 RM	SPPB	PA count	FMD	RHI
1 RM	0.69^†^					
SPPB	0.59^†^	0.47^†^				
PA Count	0.10	0.13	0.35^†^			
FMD	0.43^†^	0.11	0.37^†^	0.35^†^		
RHI	0.42^†^	0.08	0.13	0.11	0.27	
AIx	−0.54^†^	−0.32	−0.29	−0.12	−0.31	−0.28

^†^ *P* < 0.05.

**Table 3:** Correlation matrix for measures of vascular function and muscle function adjusted for potential confounders^∗^.

	40% 1 RM	1 RM	SPPB	PA count	FMD	RHI
1 RM	0.51^†^					
SPPB	0.58^†^	0.41^†^				
PA Count	0.38^†^	0.26	0.48^†^			
FMD	0.61^†^	0.16	0.38^†^	0.42^†^		
RHI	0.54^†^	0.12	0.11	0.11	0.34	
AIx	−0.43^†^	−0.29	−0.24	−0.08	−0.31	−0.32

^∗^Sex, BMI, MAP, and HR adjusted.

^†^ *P* < 0.05.
